# Comparing sensitivity to change using the 6-item versus the 17-item Hamilton depression rating scale in the GUIDED randomized controlled trial

**DOI:** 10.1186/s12888-019-2410-2

**Published:** 2019-12-27

**Authors:** Boadie W. Dunlop, Sagar V. Parikh, Anthony J. Rothschild, Michael E. Thase, Charles DeBattista, Charles R. Conway, Brent P. Forester, Francis M. Mondimore, Richard C. Shelton, Matthew Macaluso, Jennifer Logan, Paul Traxler, James Li, Holly Johnson, John F. Greden

**Affiliations:** 1 Department of Psychiatry and Behavioral Sciences, Emory University School of Medicine, 12 Executive Park Dr. NE, 3rd Floor, Atlanta, GA 30329 USA; 2 Department of Psychiatry, and National Network of Depression Centers, University of Michigan Comprehensive Depression Center, Ann Arbor, MI USA; 3 UMass Memorial Healthcare, University of Massachusetts Medical School, Worcester, MA USA; 4 The Corporal Michael Crescenz VAMC, Perelman School of Medicine of the University of Pennsylvania, Philadelphia, PA USA; 5 Department of Psychiatry and Behavioral Sciences, Stanford University School of Medicine, Stanford, CA USA; 6 Department of Psychiatry, and the John Cochran Veteran’s Administration Hospital, Washington University School of Medicine, St. Louis, MO USA; 7 McLean Hospital, Division of Geriatric Psychiatry, Harvard Medical School, Belmont, MA USA; 8 Department of Psychiatry and Behavioral Sciences, Johns Hopkins University School of Medicine, Baltimore, MD USA; 9 Department of Psychiatry and School of Medicine, The University of Alabama at Birmingham, Birmingham, AL USA; 10 Department of Psychiatry and Behavioral Sciences, University of Kansas School of Medicine-Wichita, Wichita, KS USA; 11 Assurex Health, Inc./Myriad Neuroscience, Mason, OH USA; 12 Myriad Genetics, Inc., Salt Lake City, UT USA

**Keywords:** Genetics, Antidepressant, Depression, Biomarker, Pharmacogenomics, Clinical trial, Comparative effectiveness, Clinical utility, Decision-making, Assessment

## Abstract

**Background:**

Previous research suggests that the 17-item Hamilton Depression Rating Scale (HAM-D17) is less sensitive in detecting differences between active treatment and placebo for major depressive disorder (MDD) than is the HAM-D6 scale, which focuses on six core depression symptoms. Whether HAM-D6 shows greater sensitivity when comparing two active MDD treatment arms is unknown.

**Methods:**

This post hoc analysis used data from the intent-to-treat (ITT) cohort (*N* = 1541) of the Genomics Used to Improve DEpression Decisions (GUIDED) trial, a rater- and patient-blinded randomized controlled trial. GUIDED compared combinatorial pharmacogenomics-guided care with treatment as usual (TAU) in patients with MDD. Percent of symptom improvement, response rate and remission rate from baseline to week 8 were evaluated using both scales. Analyses were performed for the full cohort and for the subset of patients who at baseline were taking medications predicted by the test to have moderate or significant gene-drug interactions. A Mokken scale analysis was conducted to compare the homogeneity of HAM-D17 with that of HAM-D6.

**Results:**

At week 8, the guided-care arm demonstrated statistically significant benefit over TAU when the HAM-D6 (∆ = 4.4%, *p* = 0.023) was used as the continuous measure of symptom improvement, but not when using the HAM-D17 (∆ = 3.2%, *p* = 0.069). Response rates increased significantly for guided-care compared with TAU when evaluated using both HAM-D6 (∆ = 7.0%, *p* = 0.004) and HAM-D17 (∆ = 6.3%, *p* = 0.007). Remission rates also were significantly greater for guided-care versus TAU using both measures (HAM-D6 ∆ = 4.6%, *p* = 0.031; HAM-D17 ∆ = 5.5%, *p* = 0.005). Patients in the guided-care arm who at baseline were taking medications predicted to have gene-drug interactions showed further increased benefit over TAU at week 8 for symptom improvement (∆ = 7.3%, *p* = 0.004) response (∆ = 10.0%, *p* = 0.001) and remission (∆ = 7.9%, *p* = 0.005) using HAM-D6. All outcomes showed continued improvement through week 24. Mokken scale analysis demonstrated the homogeneity and unidimensionality of HAM-D6, but not of HAM-D17, across treatment arms.

**Conclusions:**

The HAM-D6 scale identified a statistically significant difference in symptom improvement between combinatorial pharmacogenomics-guided care and TAU, whereas the HAM-D17 did not. The demonstrated utility of pharmacogenomics-guided treatment over TAU as detected by the HAM-D6 highlights its value for future biomarker-guided trials comparing active treatment arms.

**Trial registration:**

Clinicaltrials.gov: NCT02109939. Registered 10 April 2014.

## Background

Roughly half of patients with major depressive disorder (MDD) fail to respond to treatment with an antidepressant medication, and approximately two-thirds fail to achieve remission [1]. These inadequate outcomes have sparked great interest in exploring biological subtypes of depression that correlate with variability in medication response [2]. Pairing clearly defined subtypes with validated biomarkers such as genetic and epigenetic, proteomic, metabolomic, inflammation, neuroimaging, and electroencephalography measures might enable more precise treatment selection and response monitoring.

Genetic variation is an important biological contributor to both MDD development [3, 4] and to treatment response [5, 6]. On their own, individual gene variants explain little of the variance in disease risk or outcomes; rather, clinical manifestation of MDD and treatment response appear to result from the combined effects of many genes, along with other clinical and environmental factors. Combinatorial pharmacogenomic tests, which evaluate the weighted effects of genetic variants to predict which medications may be impacted by gene-drug interactions, hold promise for aiding patient-specific treatment selection [7]. Recently, the Genomics Used to Improve DEpression Decisions (GUIDED) randomized controlled trial (RCT) reported on the efficacy of using a combinatorial pharmacogenomic test in medication selection (guided-care), compared with treatment as usual (TAU), for patients with treatment non-responsive MDD [8]. This trial differed from traditional drug studies in that patients in both arms received active treatment. GUIDED approached but did not achieve a statistically significant difference between guided-care versus TAU for its primary outcome, percent symptom improvement at week 8 (*p* = 0.069; intent-to-treat [ITT] cohort), as assessed by the Hamilton Depression Rating Scale, 17-item (HAM-D17). However, significantly more patients achieved the secondary outcomes, response (*p* = 0.007) and remission (*p* = 0.005) at week 8, measured using HAM-D17, when they received pharmacogenomics-guided care.

The results observed in the GUIDED trial highlight the challenges in detecting clinically and statistically significant differences in randomized trials when patients in all study arms receive active treatment. This is especially true in psychiatry, where several well-powered randomized trials comparing active MDD treatments have failed to show differences in efficacy, including the Sequenced Treatment Alternatives in Depression (STAR*D) trial [9], the Genome-Based Therapeutic Drugs for Depression (GENDEP) trial [10], and the Combining Medications to Enhance Depression Outcomes COMED trial [11]. Lack of significant differences in efficacy extends even to large trials that compare psychotherapy, antidepressant medications, or their combination [12, 13]. Such equivalent outcomes, despite the treatments’ distinct mechanisms, raise the possibility that the assessment metrics used are flawed [14].

The Hamilton Depression Rating Scale (HAM-D) is the most widely used outcome measure in MDD clinical trials, with the 17-item version (HAM-D17) originally published in 1960, serving as the standard [15, 16]. Over the past four decades, however, researchers have raised concerns about the ability of the HAM-D17 scale to assess accurately the severity of and change in depression symptoms [17–19]. Factor analyses of HAM-D17 have determined that the scale is not a unidimensional measure of depression severity but rather consists of two to eight symptom factors [20]. Although multidimensionality in a scale is useful for detecting a broad array of clinical features, a multidimensional (or multifactorial) scale may reduce the ability to detect change over time, because some factors may not adequately distinguish groups when valid differences exist [21]. The ability to scale appropriately with illness severity is a fundamental aspect of construct validity. Medication side effects may affect some factors on multidimensional scales more than others, potentially producing total score changes that do not align with changes in core depressive symptoms [22, 23]. In studies such as GUIDED that allow concomitant treatments (e.g., sedative hypnotics for insomnia and anxiety in conjunction with antidepressant medication), assessing efficacy with HAMD-17 becomes even more problematic, as the uncontrolled additional medications can result in score changes unrelated to antidepressant treatment.

To address these shortcomings, researchers developed abbreviated, more focused versions of HAM-D17 [24]. Of these, the most widely used is the six-item subscale of HAM-D17, known as the HAM-D6 or melancholia subscale [23, 25]. The HAM-D6 scale is specific to the core depressive symptoms of depressed mood, guilt, work and activities, psychomotor retardation, psychic anxiety, and general somatic symptoms (energy and physical pain), and it is unidimensional [26]. HAM-D17 symptoms omitted from the HAM-D6 scale include suicidal thoughts, initial insomnia, middle insomnia, late insomnia, psychomotor agitation, somatic anxiety, gastrointestinal symptoms [appetite], sexual disturbances, hypochondriasis [somatization], insight, and weight loss. The HAM-D6 scale correlates better with the Clinical Global Impressions Scale-Severity than does the HAM-D17 scale, particularly among more severely ill patients [21]. It has repeatedly demonstrated greater effect sizes for second-generation antidepressants than has HAM-D17, as well as similar effect sizes for medications that have sedating side effects, such as TCAs and mirtazapine [27–29].

This post hoc analysis of GUIDED trial data evaluated whether the HAM-D6 scale showed significant differences in outcomes between patients whose treatment was guided by combinatorial pharmacogenomic testing versus TAU. We hypothesized that the more sensitive and unidimensional HAM-D6 would detect a statistically significant difference in symptom improvement between the guided-care and TAU arms, whereas the difference approached but did not achieve significance (*p* = 0.069) using HAM-D17. We also examined whether the statistically significantly higher rates of response and remission observed using the HAM-D17 scale would be replicated using HAM-D6.

## Methods

### Pharmacogenomic testing

All enrolled patients were tested with a combinatorial pharmacogenomic test (GeneSight Psychotropic, Assurex Health, Inc., now Myriad Neuroscience, Mason, OH). At the time of the study, the test evaluated genotypes for 59 alleles and variants across eight genes (*CYP1A2, CYP2C9, CYP2C19, CYP3A4, CYP2B6, CYP2D6, HTR2A,* and *SLC6A4*) [30]. Using a proprietary algorithm that weighted the combined influences of individual genotypes on each of 38 medications, a report was generated that categorized the medications into three levels of gene-drug interaction: ‘use as directed’ (no detected gene-drug interactions); ‘use with caution’ (moderate gene-drug interactions, i.e., medications may be effective with dose modification); and ‘use with increased caution and with more frequent monitoring’ (significant gene-drug interactions that may significantly impact drug safety and/or efficacy) [31].

### Study description

The GUIDED trial was a 24-week blinded, randomized, controlled trial that evaluated the utility of combinatorial pharmacogenomic testing in medication selection (guided-care) compared with TAU for adults with MDD. Unlike traditional drug studies, patients in both study arms received active treatment. The study was performed in primary care and psychiatry specialty clinics across 60 U.S. community and academic sites.

Patients and raters were blinded to study arm. Physicians in TAU were blinded to pharmacogenomic test results. The study protocol was approved by the Copernicus Group independent review board (INC1-14-012) and conducted in accordance with the principles of the Declaration of Helsinki and its amendments. All patients provided written informed consent for participation. Detailed methods and primary analyses for the GUIDED trial have been described previously [8]. Methods relevant to the current analysis are summarized here.

Prior to the baseline visit, patients were randomized 1:1 to the guided-care or TAU arm. Active treatment was provided to patients in both arms, with medications selected based on clinician judgment, informed by the pharmacogenomic test report for the guided-care arm, and “standard” clinician judgement in the non-guided arm. Clinicians for patients in the guided-care arm were not required to adhere to the test results in making medication decisions, and no medications were prohibited.

Patient assessments were performed at week 0 (baseline) and at the end of weeks 4, 8, 12 and 24. Patients and raters in both arms were blinded to study arm and pharmacogenomic test results. Clinicians for patients in the TAU arm were blinded to test results until after completion of the week 8 visit. Blinding of patients, sites, and physicians was maintained through week 8. Sites were instructed to unblind patients to their randomization assignment following the week 12 assessment. Because patient unblinding may have occurred before week 12 assessments were performed, however, only data collected through the week 8 assessment were considered blinded.

### Participants

Patients were enrolled if they were diagnosed with DSM-IV-TR-defined MDD, confirmed by both the self-rated and site-rated 16-item Quick Inventory of Depression Symptomology (QIDS-SR16 and QIDS-C16 ≥ 11) at screening and baseline, and if they reported an inadequate response within the current depressive episode to at least one medication included on the pharmacogenomic test report. Key exclusion criteria included significant short-term suicide risk, bipolar disorder, current delirium or neurocognitive disorder, psychotic disorder or psychotic symptoms during the current or a previous depressive episode, a current substance use disorder, or a significant unstable medical condition.

### Statistical analysis

Analyses described herein were conducted using the ITT cohort, which included all patients who met eligibility criteria, were randomized to a study arm, and had at least one post-baseline visit. Outcomes analyses were performed for the ITT cohort and separately for the subset of patients who at baseline were taking medications predicted to have moderate or significant gene-drug interactions (those in the ‘use with caution’ and ‘use with increased caution and more frequent monitoring’ report categories). This subset excluded patients who were taking only medications in the ‘use as directed’ category.

The protocol-defined primary efficacy measure for GUIDED was the HAM-D17 scale, administered by blinded central raters (MedAvante-ProPhase Inc., Hamilton, NJ). For this post hoc scale comparison, HAM-D6 scores were derived from the HAM-D17 assessments. These included: item 1, depressed mood; item 2, guilt feelings; item 7, work and activities; item 8, psychomotor retardation; item 10, psychic anxiety; and item 13, general somatic symptoms. Items 1, 2, 7, 8, and 10 each were scored from 0 to 4, and item 13 was scored from 0 to 2, for a maximum possible HAM-D6 score of 22. For HAM-D17, the maximum possible score was 52.

The primary endpoint was percent *symptom improvement* from baseline to week 8, and secondary endpoints were *response* and *remission* rates at week 8. *Response* was defined as a ≥ 50% decrease in score at week 8 from baseline and was assessed for both HAM-D17 and HAM-D6. *Remission* was defined as having a score of ≤7 for HAM-D17 [32] and ≤ 4 for HAM-D6 [21, 33]. The durability of pharmacogenomic testing utility was evaluated in the guided-care arm through outcome assessments at weeks 4, 8, 12, and 24.

Identical statistical methods were used for the primary HAM-D17 analyses and the post hoc HAM-D6 analyses. A mixed model for repeated measures was used to assess percent change in symptoms from baseline to week 8. A generalized linear mixed model was used for separate analyses of response and remission at week 8. Because the response and remission outcomes were measured at both week 4 and week 8, a generalized linear mixed model (GLMM) was used to account for both within-subject and between-subject variability over time. Both the mixed model for repeated measures and the GLMM included treatment, week, treatment-by-week interaction, baseline HAM-D6 score, and baseline HAM-D6 score-by-week interaction as fixed effects. Binomial distribution with a log-link function was used for the GLMM model. The pairwise comparisons between the two treatment arms at week 8 were tested at a significance level of 0.05 (2-sided). Missing values were handled using maximum likelihood method via mixed models for repeated measures for both symptom improvement and via generalized linear mixed model for categorical variables – response and remission. Analyses were performed with SAS software (version 9.4) or JMP 14 (SAS Institute).

An analysis of scalability was performed using the non-parametric item response theory model developed by Mokken [34]. Using this framework, the deviation of either the HAM-D17 scale or the HAM-D6 scale from a perfectly homogeneous structure was expressed using Loevinger’s scalability coefficient (H) [35], a measure of the extent to which the scale items represented a single dimension. Loevinger’s coefficient was interpreted as follows: ≥0.5, strong scale homogeneity; 0.40–0.49, moderate but acceptable homogeneity; 0.30–0.39, doubtful homogeneity; < 0.30, no homogeneity.

## Results

### Cohort description

At baseline, the ITT cohort included 1541 patients (guided-care, *n* = 760; TAU, *n* = 781). Baseline clinical characteristics of the cohort are presented in Table 1. There were no meaningful differences between the two treatment arms in depression characteristics, HAM-D17 scores or HAM-D6 scores at baseline. At the week 8 time point, the ITT cohort included 1298 patients (guided-care, *n* = 621; TAU, *n* = 677).

**Table 1:** Clinical features of the GUIDED intent-to-treat study population at baseline (week 0)

	Treatment		
Characteristic	TAU (*N* = 781)	Guided-Care (N = 760)	Total (N = 1541)
HAM-D17			
Mean (SD)	20.74 (4.86)	20.49 (4.84)	20.62 (4.85)
Min, Max	6.0, 35.0	4.0, 37.0	4.0, 37.0
Depression Category, n (%)			
None (0–7)	5 (0.6)	6 (0.8)	11 (0.7)
Mild (8–13)	46 (5.9)	45 (5.9)	91 (5.9)
Moderate (14–18)	189 (24.2)	209 (27.5)	398 (25.8)
Severe (19–22)	270 (34.6)	241 (31.7)	511 (33.2)
Very Severe (≥ 23)	271 (34.7)	259 (34.1)	530 (34.4)
HAM-D6			
Mean (SD)	10.90 (2.16)	10.99 (2.11)	10.94 (2.13)
Min, Max	1.0, 17.0	3.0, 18.0	1.0, 18.0
Failed Medication Trials			
Mean (SD)	3.54 (3.01)	3.45 (3.02)	3.49 (3.01)
Min, Max	1, 34	1, 25	1, 34
Psychiatric Comorbidities, n (%)			
General anxiety disorder	104 (13.4)	127 (16.7)	231 (15.0)
Panic disorders/social phobia	112 (14.4)	119 (15.7)	231 (15.0)
Post-traumatic stress disorder	35 (4.5)	41 (5.4)	76 (4.9)

*SD* standard deviation, *TAU* treatment as usual

### Symptom improvement, response and remission: HAM-D6 versus HAM-D17

**Fig. 1:**
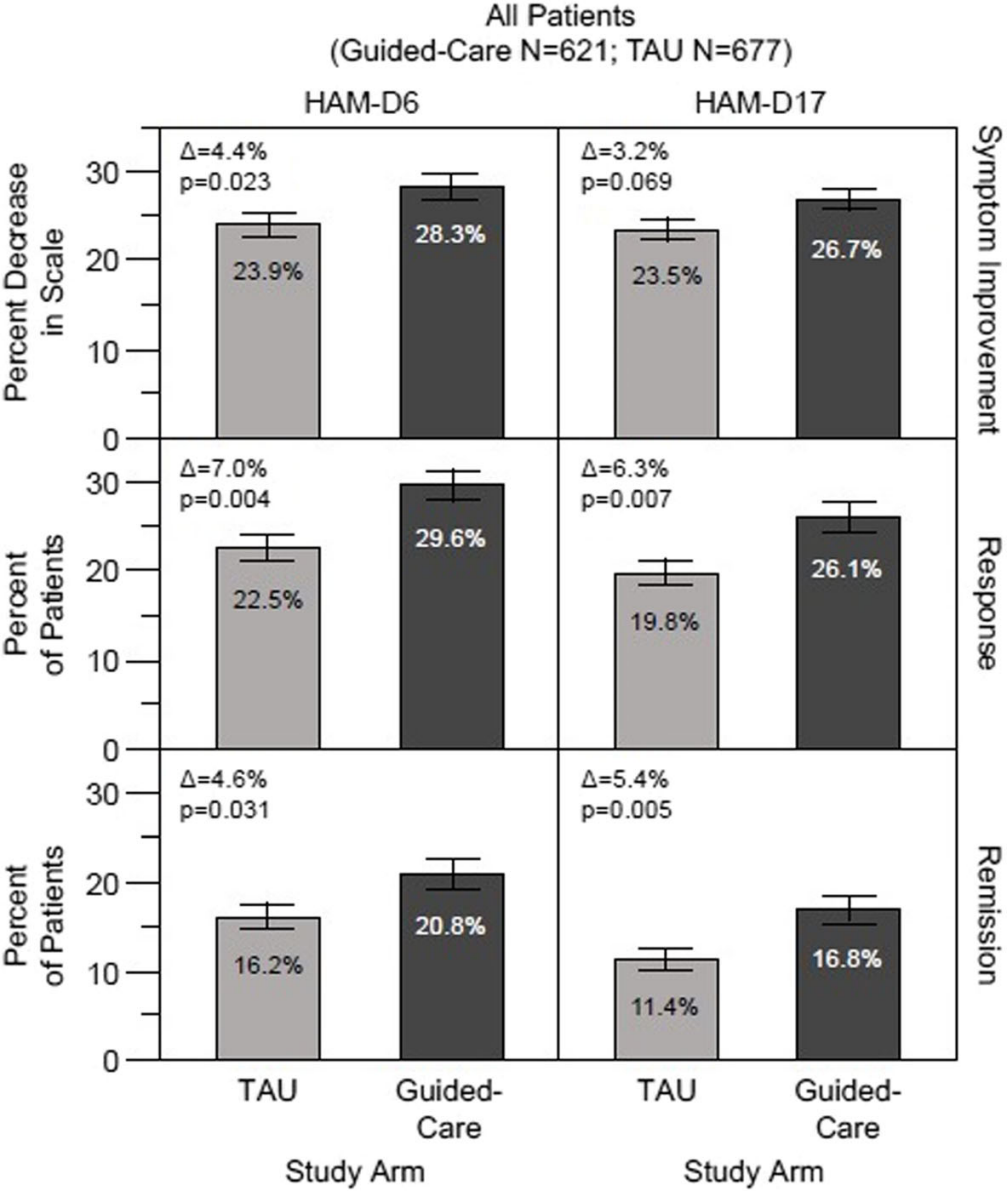
Outcomes at week 8 for the full patient cohort. The pharmacogenomics guided-care arm (*N* = 621) was compared with treatment as usual (TAU) (*N* = 677). Symptom improvement, response and remission outcomes were evaluated using the HAM-D6 and HAM-D17 depression rating scales

At week 8, there was a 28.3% decrease in HAM-D6 scores from baseline in the guided-care arm, compared with a 23.9% decrease in the TAU arm (Fig. 1). This difference in mean percent symptom improvement between arms was statistically significant (∆ = 4.4%, *p* = 0.023) compared to that reported previously using the HAM-D17 scale (∆ = 3.2%, *p* = 0.069). The response rate at 8 weeks among patients in the guided-care arm (29.6%) similarly showed a significant increase over TAU (22.5%) using HAM-D6 (∆ = 7.0%, *p* = 0.004) (Fig. 1). The percent difference between study arms also was statistically significant for HAM-D17 (∆ = 6.3%, *p* = 0.007). Remission rates at week 8 favored pharmacogenomics-guided treatment (20.8%) versus TAU (16.2%) at week 8 using HAM-D6 (Fig. 1), and the percent difference between study arms was statistically significant for HAM-D6 (∆ = 4.6%, *p* = 0.031). The remission rate in the guided-care versus TAU arms was significant using the HAM-D17 scale (∆ = 5.4%, *p* = 0.005). Overall, the results for response rate and remission rate were similar for both scales.

### Patients entering on medications with predicted gene-drug interactions

**Fig. 2:**
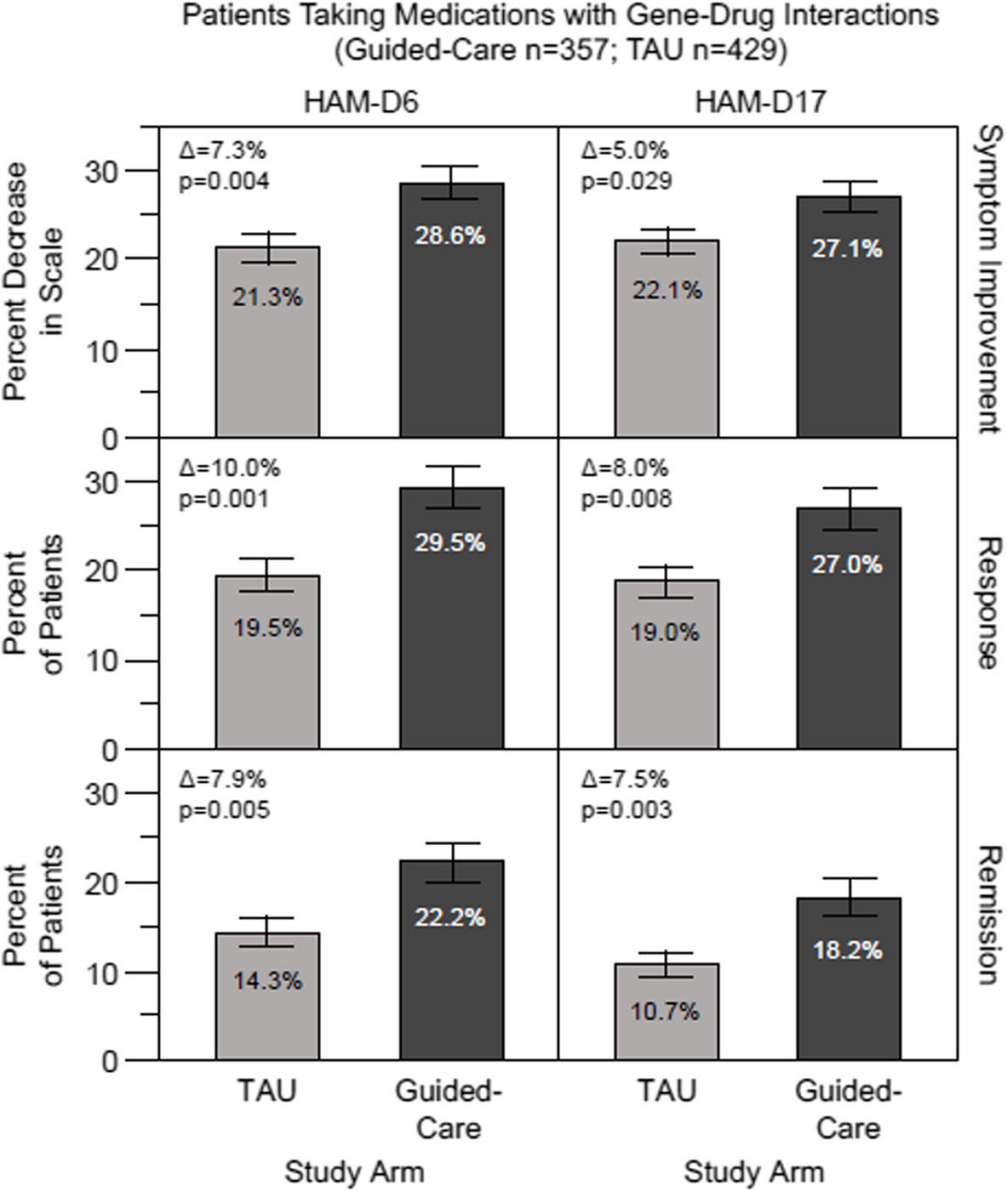
Outcomes at week 8 for patients taking medications with gene-drug interactions. The pharmacogenomics guided-care arm (*n* = 357) was compared with treatment as usual (TAU) (*n* = 429). Symptom improvement, response and remission outcomes were evaluated using the HAM-D6 and HAM-D17 depression rating scales

To examine the impact of guided-care versus TAU more specifically for patients who stand to benefit most from pharmacogenomic testing, HAM-D6 outcomes were assessed in the subset of patients who at baseline were prescribed medications predicted by the patient’s test results to have gene-drug interactions (Fig. 2). At week 8, the mean percent symptom improvement in the guided-care arm (28.6%) was significantly greater than that measured in TAU (21.3%) (∆ = 7.3%, *p* = 0.004). Response rate in the guided-care arm (29.5%) also was significantly improved over TAU (19.5%) (∆ = 10.0%, *p* = 0.001). Finally, remission rate was improved for guided-care (22.2%) versus TAU (14.3%) in these patients (∆ = 7.9%, *p* = 0.005). Compared with the outcomes assessed using the HAM-D17 scale in this subset of patients (Fig. 2) [36], the HAM-D6 scale showed equal or greater sensitivity to detect differences between guided-care and TAU for all three depression outcomes. In addition, the percent differences between guided-care and TAU across all three outcomes were substantially higher in patients predicted to be most impacted by gene-drug interactions than were those observed in the full patient cohort using either HAM-D17 or HAM-D6 (Fig. 1).

### Scale homogeneity

To assess the dimensionality of the HAM-D17 and HAM-D6 assessments in the GUIDED ITT cohort, a Mokken scale analysis was performed. Table 2 shows the Loevinger’s coefficient of homogeneity (H) at week 8 for each assessment scale. For the combined treatment arms, HAM-D17 had a coefficient of 0.30, indicating that the scale is heterogeneous and multidimensional. In contrast, HAM-D6 had a coefficient of 0.53 for the combined arms, indicating that the scale is homogeneous and unidimensional. Similar results were observed for individual treatment arms.

**Table 2:** Mokken scale analysis of homogeneity of HAM-D17 and HAM-D6 scores at week 8

Treatment Arm	N	HAM-D17 (H)	HAM-D6 (H)
Combined	1298	0.30	0.53
Guided-Care	621	0.34	0.56
TAU	677	0.27	0.51

Loevinger’s coefficient (H) values were determined for combined treatment arms, guided-care and treatment as usual. HAM-D6, Hamilton depression rating scale, 6-item; HAM-D17, Hamilton depression rating scale, 17-item; *TAU* treatment as usual

### Durability of response

**Fig. 3:**
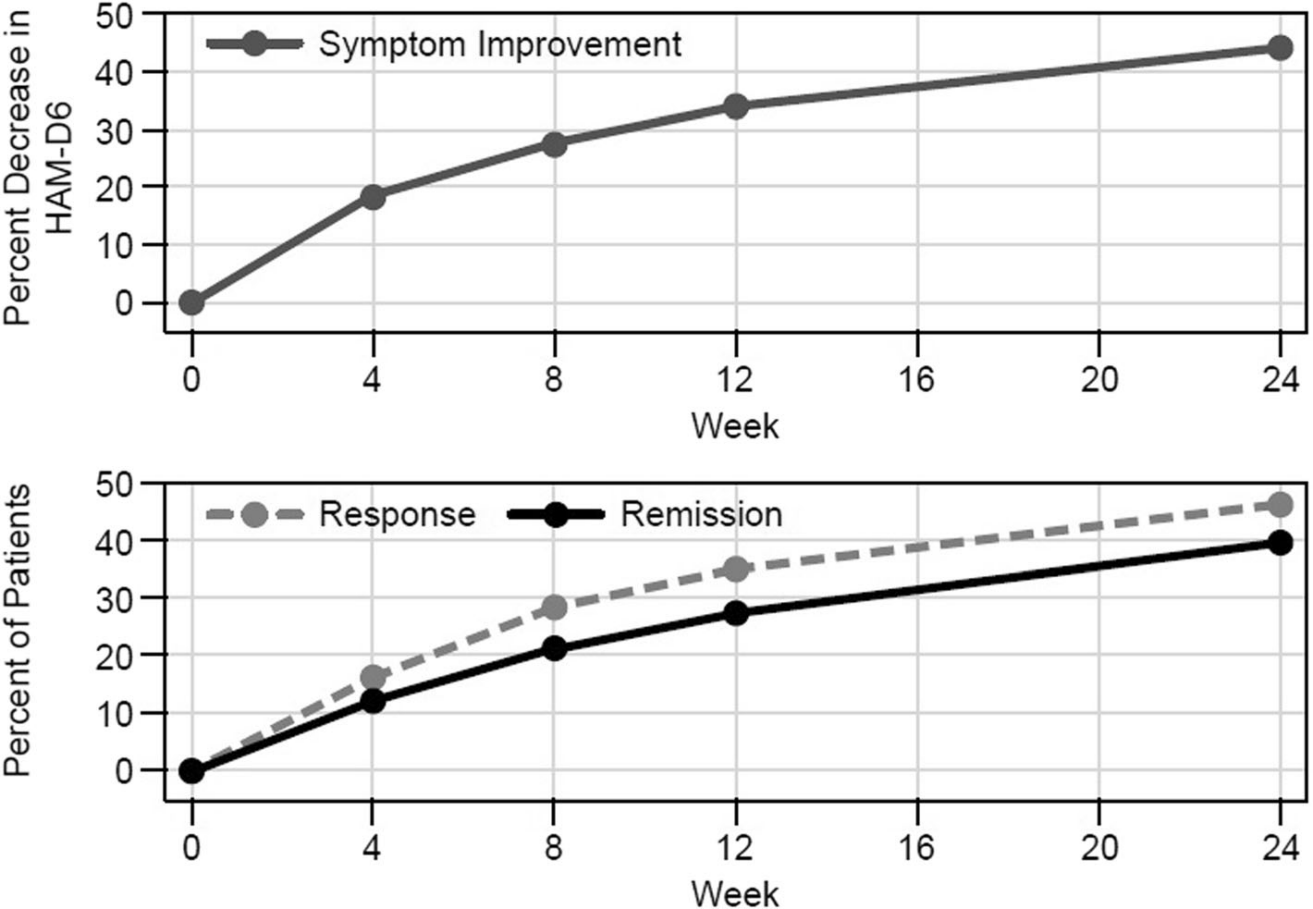
Durability of improvements in patient outcomes in the pharmacogenomics guided-care study arm. Symptom improvement, response and remission outcomes were evaluated at week 4 (*N* = 685), week 8, (*N* = 621), week 12 (*N* = 585), and week 24 (*N* = 522) using the HAM-D6 depression rating scale

To evaluate the durability of the guided-care treatment results, patient HAM-D6 scores in the guided-care arm were evaluated at time points extending through the end of the 24-week trial period (Fig. 3). Consistent increases were observed for all three measured outcomes from baseline through weeks 4, 8, 12, and 24.

## Discussion

This comparative, post hoc analysis of the HAM-D6 and HAM-D17 depression scales in the GUIDED trial for MDD treatment found greater sensitivity to differences in treatment effects with the abbreviated version of the scale. This result likely is due to the narrower focus of the HAM-D6 scale, as compared with HAM-D17, for the core symptoms of depression. Furthermore, although both versions of the scale achieved statistically significant differences for the response and remission outcomes, the greater differences for symptom improvement seen with the HAM-D6 scale suggest that HAM-D6 provided a more precise measure for MDD outcome assessment. This is supported further by the observation of the high sensitivity of the HAM-D6 scale in the subset of patients who entered the trial on medications predicted by the pharmacogenomic test to have gene-drug interactions. Mokken scale analysis further supported the increased homogeneity of HAM-D6 relative to HAM-D17. Altogether, these results mirror those seen in many placebo-controlled pharmacological trials, wherein the HAM-D17 scale failed to identify an antidepressant effect, while the HAM-D6 scale did [27].

Although the percent differences in response and remission rates were generally similar for HAM-D17 and HAM-D6, the slightly lower difference in remission rate between arms (0.8%) as assessed by the HAM-D6 (∆ = 4.6%) versus the HAM-D17 (∆ = 5.4%) is of interest. A concern in the field is that the standard HAM-D17 remission threshold of ≤7 may be high, capturing many patients who continue to experience impairment or distress from persisting symptoms [35, 36]. Thus, low levels of core symptoms, as determined by the standard HAM-D6 remission threshold (≤4), might comprise a more valid measure for defining the state of clinical remission. The question of whether the HAM-D6 or the HAM-D17 remission threshold better predicts restoration of function and long-term wellness should be a focus of future work.

The importance of maximizing signal detection through use of the most sensitive scale to detect treatment effects is of particularly great importance for comparative effectiveness studies and for biomarker-based clinical trials, both of which provide active treatment to all patients [37]. Over the past several decades, adequately powered MDD trials comparing active treatments, be they medications or psychotherapies, have found no difference between treatment arms [9–11]. Notably, all of these large trials have used either the HAM-D17 scale, the Montgomery-Åsberg Depression Rating Scale (MADRS), or the Quick Inventory of Depressive Symptomatology-Self-Report (QIDS-SR) as the efficacy measure, each of which contains numerous items unrelated to core depression symptoms captured by the HAM-D6 scale. Trials such as GUIDED that allow concomitant medications for specific symptoms, such as sedative hypnotics for anxiety or insomnia, can further diminish the ability to identify a difference between treatment arms when the outcome measure includes non-core depressive symptoms [38]. Consequently, future randomized trials applying biomarker-based approaches to treatment selection for MDD may benefit from using the HAM-D6 or a similar, more focused symptom scale.

The greater discriminative ability of the HAM-D6 scale also allows for smaller sample sizes to test hypotheses about efficacy [39–41]. Given the greater precision and numerous advantages of HAM-D6, it is difficult to justify continued use of the full HAM-D17 scale as the sole primary outcome measure in MDD treatment trials. In the future, HAM-D17 could be used to enable historic comparisons of baseline severity among trials, but new study protocols should consider specifying the HAM-D6 or a similarly more precise assessment of core symptoms [42, 43] as the primary efficacy variable for analysis. Administering the shorter version can have the added benefit of reducing time burdens on clinical trial participants.

This analysis had several strengths that were inherent to the GUIDED primary analysis. First, the diversity of the study cohort mirrors that seen across varied clinical scenarios for MDD treatment, including clinicians in both psychiatric specialty and primary care clinics. Second, the study’s two active treatment arms reflect real-world clinical practice and provide a relevant evaluation of clinical utility. The limitations of the primary GUIDED analysis also apply to this study [8]. Specifically, the treating clinician was not blinded to study arm, though this limitation was mitigated somewhat by using blinded central raters, and by keeping the site raters and patients blinded to study arm until after week 8. The impact of polypharmacy is another intrinsic limitation; however, as was discussed in the primary analysis, confounding effects likely would be equivalent between study arms. A specific limitation of using the HAM-D6 scale is that it does not assess for some important depressive symptoms, including physical symptoms [24] and suicide. The routine use of separate, more comprehensive suicide assessments in modern clinical trials of MDD treatments reduces concern about this limitation.

## Conclusion

The results of this analysis are consistent with a substantial body of published evidence showing that HAM-D6, which is focused more precisely on core depressive symptoms, is more sensitive than HAM-D17 in assessing depression symptom improvement in patients with MDD. The demonstrated utility of pharmacogenomics-guided treatment over TAU as detected by the HAM-D6 in the GUIDED trial highlights its value for future biomarker-guided trials comparing multiple active treatment arms.

## Data Availability

All data generated or analyzed during this study are included in this published article.

## Abbreviations

GUIDED: Genomics Used to Improve DEpression Decisions trial
HAM-D: Hamilton Depression Rating Scale
MADRS: Montgomery-Åsberg Depression Rating Scale
MDD: major depressive disorder
QIDS-SR: Quick Inventory of Depressive Symptomatology-Self-Report
TAU: treatment as usual
